# Dietary Deoxynivalenol Contamination and Oral Lipopolysaccharide Challenge Alters the Cecal Microbiota of Broiler Chickens

**DOI:** 10.3389/fmicb.2018.00804

**Published:** 2018-04-25

**Authors:** Annegret Lucke, Josef Böhm, Qendrim Zebeli, Barbara U. Metzler-Zebeli

**Affiliations:** Department for Farm Animals and Veterinary Public Health, Institute of Animal Nutrition and Functional Plant Compounds, University of Veterinary Medicine Vienna, Vienna, Austria

**Keywords:** broiler, 16S RNA sequencing, cecum, deoxynivalenol, lipopolysaccharide, microbiota

## Abstract

Dietary deoxynivalenol (DON) impairs the intestinal functions and performance in broiler chickens, whereas little is known about the effect of DON on the gastrointestinal microbiota. This study evaluated the impact of graded levels of dietary DON contamination on the cecal bacterial microbiota, their predicted metabolic abilities and short-chain fatty acid (SCFA) profiles in chickens. In using a single oral lipopolysaccharide (LPS) challenge we further assessed whether an additional intestinal stressor would potentiate DON-related effects on the cecal microbiota. Eighty 1-day-old chicks were fed diets with increasing DON concentrations (0, 2.5, 5, and 10 mg DON per kg diet) for 5 weeks and were sampled after half of the chickens received an oral LPS challenge (1 mg LPS/kg bodyweight) 1 day before sampling. The bacterial composition was investigated by Illumina MiSeq sequencing of the V3–5 region of the 16S rRNA gene. DON-feeding decreased (*p* < 0.05) the cecal species richness (Chao1) and evenness (Shannon) compared to the non-contaminated diet. The phyla *Firmicutes* and *Proteobacteria* tended to linearly increase and decrease with increasing DON-concentrations, respectively. Within the *Firmicutes*, DON decreased the relative abundance of *Oscillospira, Clostridiaceae* genus, *Clostridium*, and *Ruminococcaceae* genus 2 (*p* < 0.05), whereas it increased *Clostridiales* genus 2 (*p* < 0.05). Moreover, increasing DON levels linearly decreased a high-abundance *Enterobacteriaceae* genus and an *Escherichia/Shigella*-OTU (*p* < 0.05). Changes in the bacterial composition and their imputed metagenomic capabilities may be explained by DON-related changes in host physiology and cecal nutrient availability. The oral LPS challenge only decreased the abundance of an unassigned *Clostridiales* genus 2 (*p* = 0.03). Increasing dietary concentrations of DON quadratically increased the cecal total SCFA and butyrate concentration (*p* < 0.05), whereas a DON × LPS interaction indicated that LPS mainly increased cecal total SCFA, butyrate, and acetate concentrations in chickens fed the diets that were not contaminated with DON. The present findings showed that even the lowest level of dietary DON contamination had modulatory effects on chicken's cecal bacterial microbiota composition and diversity, whereas the additional oral challenge with LPS did not potentiate DON effects on the cecal bacterial composition.

## Introduction

The gut microbiota plays a crucial role in host health through various functions like vitamin synthesis, digestion of dietary fiber, modulation of the gut epithelial barrier, and inflammatory responses as well as protecting against colonization with pathogens (Maslowski and Mackay, 2011; Kogut and Arsenault, 2016). In chickens, the ceca are the gut site with the highest microbial colonization (Oakley et al., 2014), with *Bacteroidaceae, Ruminococcaceae, Lachnospiraceae*, and *Clostridiaceae* being the dominating bacterial taxa (Oakley et al., 2014). Moreover, the longer retention time of digesta in the ceca allows for a more complete microbial breakdown of complex fiber and enhances short-chain fatty acid (SCFA) production compared to the other gut sites (Oakley et al., 2014).

Because of the widespread contamination of cereal grains with the *Fusarium* toxin deoxynivalenol (DON) combined with the vast use of cereal grains in livestock diets worldwide, DON-contaminated feed is a very harmful factor for animal health (Escrivá et al., 2015), and causes substantial economic losses in poultry production (Awad et al., 2013). Chickens are believed to be sensitive to moderate DON levels that compromise growth performance (Andretta et al., 2011) and functioning of the immune system (Awad et al., 2013). The current guidance value of the European Union that is applicable for complete feed for poultry is 5 mg DON/kg feed (12% moisture) (2006/576/EC, 2006). Consumption of DON impairs the intestinal morphology, nutrient absorption, barrier function, and the innate immune response in chickens (Awad et al., 2011b; Osselaere et al., 2013), whereas the interaction between DON and the chicken gut microbiota has been hardly elucidated (Ghareeb et al., 2015). Evidence from other species (e.g., pigs, human microbiota-associated rats) demonstrates that exposure to DON modifies the gut bacterial community (Waché et al., 2009; Saint-Cyr et al., 2013; Piotrowska et al., 2014). Accordingly, DON increased the genera *Bacteroides* and *Prevotella* while decreasing *Escherichia coli* in a human microbiota-associated rat model (Saint-Cyr et al., 2013). Moreover, the gut microbiota has been reported to metabolize mycotoxin compounds, which may alter the identity and toxicity of metabolites for the host (Gratz et al., 2013, 2017). Due to the role that the gut microbiota plays in priming of gut functions and immune development (Schokker et al., 2015), DON-induced alterations in the gut bacterial composition and microbe-host interactions may account for some of the adverse effects reported for DON (Robert et al., 2017).

The gut microbiota itself releases immune-stimulatory compounds, such as lipopolysaccharides (LPS) (Saadia et al., 1990; Ge et al., 2000; Ghareeb et al., 2016), which are part of the outer membrane of Gram negative bacteria and may depress growth performance in poultry by diverting energy for an elevated immune response (Ghareeb et al., 2016). Oral challenges with LPS have been reported to cause gut mucosal tissue damage (Wu et al., 2013) and modify mucus secretion and composition *in vitro* (Dohrman et al., 1998; Smirnova et al., 2003; Cornick et al., 2015; Zhang et al., 2017). Moreover, evidence suggests that LPS can interfere with the response to other xenobiotic agents. In doing so, LPS has been shown to increase the toxicity of trichothecenes such as DON in rodent models (Zhou et al., 2000; Ganey and Roth, 2001; Islam and Pestka, 2006). Damage to the gut barrier in duodenum and jejunum may modify intestinal nutrient flows with consequences for the microbial composition and metabolism in the ceca. Therefore, gaining knowledge about potential interactions between common stressors, such as DON and LPS, on gut microbial profiles may help in the development of effective strategies to reduce their deleterious effects in broiler production.

The main objective of the present study was to investigate the effects of a chronic exposure to graded levels of dietary DON contamination on the cecal bacterial microbiota, their predicted metabolic functions and SCFA profile in broiler chickens. Our hypothesis was that the exposure to increasing levels of DON from the first day of life would alter the bacterial community composition and metabolic capabilities in growing chickens, leading to alterations in metabolic fermentation profile and host performance and health. In using a single oral LPS-challenge we further aimed to assess whether an additional stressor for the gut would potentiate DON-related effects on the cecal microbiota. The dietary levels of DON used in this study were chosen on the basis of the current guidance values in the European Union and are therefore of practical relevance. Data of feed intake and performance can be found in our companion paper (Lucke et al., 2017a).

## Materials and methods

### Ethics statement

The animal procedures were approved by the institutional ethics committee of the University of Veterinary Medicine Vienna and the Austrian national authority according to paragraph 26 of Law for Animal Experiments, Tierversuchsgesetz 2012—TVG 2012 (GZ 68.205/0062–WF/V/3b/2015).

### Experimental design

The feeding experiment, housing conditions and diets are described in detail in Lucke et al. (2017a). Briefly, a total of 80 one-day-old broiler chicks (ROSS 308) obtained from a commercial hatchery (Brüterei Schlierbach GmbH, Pettenbach, Austria) were randomly allocated to four feeding groups: (1) control diet without DON (0 DON), (2) control diet experimentally contaminated with 2.5 mg DON/kg diet (2.5 DON; Romer Labs, Tulln, Austria; Supplementary Table S1), (3) control diet with 5 mg DON/kg diet (5 DON), and (4) control diet with 10 mg DON/kg diet (10 DON). The natural DON contamination in the control diet amounted to 0.16 mg/kg diet (Lucke et al., 2017a). The diets were fed from the first day of life until the end of the experimental period (d 34–37 of life). The chickens were housed in flatdeck cages (0.36 m^2^ each) in groups of 2–4 animals per cage from week 1 to 3 of the experiment and 1–2 birds per cage for the remaining time of the experiment (Lucke et al., 2017a,b). Cage floors were covered with clean cardboard paper every 2 days. One day before (d 33–36) cecal digesta collection, half of the chickens orally received 1 mg LPS per kg body weight (LPS L2880 from *E. coli* O55:B5, Sigma-Aldrich, St. Louis, Missouri, USA) dissolved in distilled water, via crop gavage, whereas the other half received distilled water as placebo (negative control). This resulted in the following eight treatment groups: 0 DON+con, 0 DON+lps, 2.5 DON+con, 2.5 DON+lps, 5 DON+con, 5 DON+lps, 10 DON+con, 10 DON+lps. On d 34–37, chickens were euthanized by an overdose of Thiopental (50–100 mg/kg body weight medicamentum pharma GmbH, Allerheiligen im Mürztal, Austria) into the wing vein followed by exsanguination. Immediately thereafter, the entire intestine was carefully removed from the abdomen and the ceca were separated. The ceca were opened longitudinally and cecal digesta were collected, homogenized with a sterile spatula, transferred to CryoPure Tubes (Sarstedt AG + Co., Nümbrecht, Germany), snap-frozen in liquid nitrogen and stored at −80°C until analysis.

### Genomic DNA isolation and 16S rRNA gene sequencing

Total DNA was isolated from 250 to 300 mg of frozen cecal digesta using the Power Soil DNA Isolation Kit (MoBio Laboratories Inc., Carlsbad, CA, USA) with few modifications as described previously (Metzler-Zebeli et al., 2016). The isolated DNA was stored at −20°C. The DNA concentration was measured using the Qubit 2.0 Fluorometer (Life Technologies, Carlsbad, CA, USA) and the Qubit dsDNA HS Assay Kit (Life technologies, Carlsbad, CA, USA).

The 16S rRNA gene PCR of the V3–V5 hypervariable region (primer set 357F_hmp (CCTACGGGAGGCAGCAG) and 926R_hmp (CCGTCAATTCMTTTRAGT), product length ~570 bp), library preparation and Illumina MiSeq sequencing were carried out by Microsynth AG (Balgach, Switzerland) (Metzler-Zebeli et al., 2015). Libraries were constructed by ligating sequencing adapters and indices onto purified PCR products using the Nextera XT sample preparation kit (Illumina Inc., San Diego, CA, USA) according to the recommendations of the manufacturer. For each of the libraries, equimolar amounts were pooled and sequenced on an Illumina MiSeq Personal Sequencer using a 300 bp read length paired-end protocol. The resultant overlapping paired-end reads were demultiplexed, trimmed using cutadapt (Martin, 2011; https://cutadapt.readthedocs.org/), stitched using Fast Length Adjustment of SHort reads (FLASH; http://www.cbcb.umd.edu/software/flash; Magoč and Salzberg, 2011) by Microsynth. In total, 3,627,930 stitched reads for the 80 cecal samples with a mean Phred score of 34–36 were obtained from the commercial provider.

### Bioinformatic analysis

Sequencing data were analyzed using QIIME (version 1.9.1; Siegerstetter et al., 2017). Fastq files were quality filtered using a quality score of 15. Chimera were detected and removed by the UCHIME method using the 64-bit version of USEARCH and the GOLD database (drive5.com; Edgar, 2010; Edgar et al., 2011). Open-reference operational taxonomic unit (OTU) picking was performed at 97% similarity level using UCLUST (Edgar, 2010). OTU taxonomy was assigned against the Greengenes database (gg_13_8; http://qiime.org/home_static/dataFiles.html). Rare OTUs with less than 10 sequences were removed. After quality control and removal of chimeras, 2,860,912 sequences remained, with a mean of 35,761 reads per sample and mean read length of 536 bp which were classified into 2,989 OTUs. For α-diversity analyses a rarefaction depth of 17,500 sequences per sample was used. OTUs being differently abundant between treatments were additionally checked using the Greengenes database (http://greengenes.lbl.gov). Proposed species obtained by BLAST sharing the highest percentage of similarity were reported. The raw sequence reads were uploaded to the NCBI BioProject databank under the project ID: PRJNA419703.

Microbial function prediction for each cecal sample based on 16S rRNA gene sequencing data was determined using Phylogenetic Investigation of Communities by Reconstruction of Unobserved States (PICRUSt; Langille et al., 2013). For this, closed-reference OTU picking was performed at 97% similarity level against the Greengenes database (downloaded from http://greengenes.secondgenome.com/downloads/database/13_5), clustered based on a 0.03 distance limit, and processed in the online Galaxy PICRUSt interface (http://galaxyproject.org/) with a workflow described by the developers (http://picrust.github.com/picrust/tutorials/quickstart.html#quickstartguide). Sequences were categorized by function based on cluster of orthologous groups of proteins (COG) and Kyoto Encyclopedia of Genes and Genomes (KEGG) pathways in PICRUSt. Non-bacteria related COG and KEGG orthology functions were dismissed.

### Microbial fermentation

The SCFA (i.e., acetic acid, propionic acid, isobutyric acid, n-butyric acid, isovaleric acid, n-valeric acid, and caproic acid) in cecal digesta were determined using gas chromatography (GC). For this, 0.5 g of cecal digesta was mixed with 0.5 g H_2_0 (double-distilled), 200 μl phosphoric acid (25%), and 300 μl of an internal standard (4-methylvalerian acid). Samples were vortexed and centrifuged at 16,300 × g for 20 min. The supernatant was collected into a fresh tube. Centrifugation steps were repeated until the supernatant became clear. Afterwards, the supernatant was stored at −80°C and analyzed on the GC (Fisons GC Model 8060 MS DFPC, Rodano, Italy) equipped with a 30 m × 0.53 mm × 0.5 μm capillary column, a flame-ionization detector (Fisons EL980) and an autosampler (Fisons autosampler Modell AS 800 C.U.). Helium was used as carrier gas (flow rate: 2 ml/min) and the temperatures of the injector and detector were set at 170 and 190°C, respectively. The GC oven program was defined as follows: The starting temperature was set at 65°C and was heated with a heating rate of 15 K/min to 170°C. The program continued with a heating rate of 35 K/min to 190°C and afterwards with a heating rate of 40 K/min to 200°C.

### Statistical analysis

The Shapiro-Wilk test was firstly used to test for normality of data distribution for all variables using the PROC UNIVARIATE procedure in SAS (version 9.4; SAS Inst. Inc., Cary, NC). After establishing normality for all data, relative bacterial abundances, relative predicted microbial function abundances and SCFA concentrations were analyzed by ANOVA using the MIXED procedure of SAS. DON treatment, LPS and their interaction were considered as fixed effects. The experimental run was included as random effect with chicken nested within group and day as the experimental unit. Orthogonal contrasts were used to test linear and quadratic relationships between control feeding and the 3 increasing levels of DON as well as the overall difference of 0 DON vs. all DON groups. Degrees of freedom were approximated using Kenward-Rogers method (ddfm = kr). Least squares means and the standard error of the mean are presented. Pairwise comparisons among least squares means were performed using the probability of difference (pdiff) option in SAS. Significance was declared if *p* < 0.05 and a trend was reported if *p* < 0.10. Fold-changes were calculated by dividing the difference between a treatment and the control by the value of the respective control. Pearson's correlations were calculated with the CORR procedure of SAS. Only significant correlations are reported and correlation matrices were visualized using the corrplot package in R Studio version 3.4.1 (Wei and Simko, 2016).

## Results

### Overall bacterial community composition

**Figure 1:**
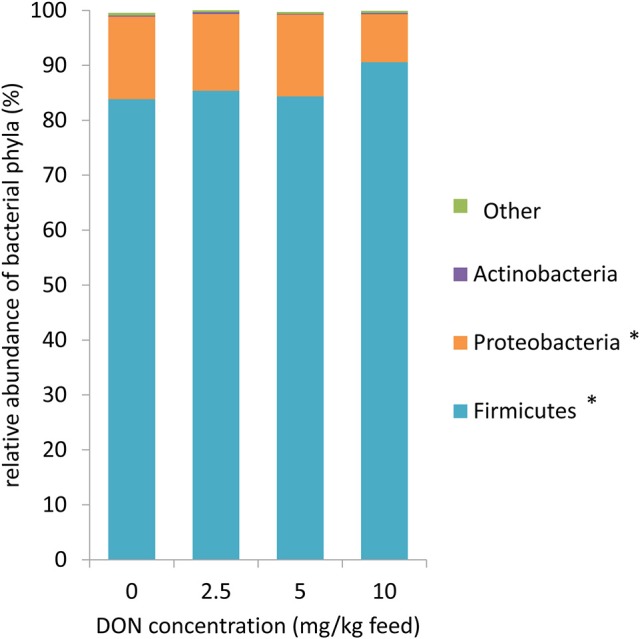
Microbiome composition at phylum level for 16S rRNA sequences in cecal digesta of broiler chickens fed diets with increasing levels of deoxynivalenol (DON; 0, 2.5, 5, or 10 mg DON/ kg diet). Values are presented as least squares means ± standard error of the mean (SEM); *n* = 20 broilers per feeding group. Presented data include both animals with and without oral lipopolysaccharide challenge (LPS) 1 day prior to slaughter within the respective feeding group. Data were not affected by LPS and DON × LPS (*p* > 0.10). ^*^ Linear contrast: *p* < 0.10.

Across all treatment groups (Figure 1), *Firmicutes* (86.0%) and *Proteobacteria* (13.2%) were the most abundant phyla in cecal digesta. At family level, the cecal community was dominated by an unassigned family of the order *Clostridiales* (38.1%) and *Ruminococcaceae* (30.6%) followed by *Enterobacteriaceae* (13.2%), *Turicibacteriaceae* (8.8%), and *Lachnospiraceae* (7.0%) (Table 1). Correspondingly, the predominating genera were an unassigned genera of the order *Clostridiales* (38.1%), *Ruminococcaceae* (20.7%), and *Enterobacteriaceae* (12.5%, largely represented by the *Escherichia/Shigella* OTU2; Table 2, Supplementary Tables S2, S3), *Turicibacter* (8.8%), *Ruminococcus* (5.8%), an unassigned genus of the family *Lachnospiraceae* (3.0%), and *Oscillospira* (2.4%).

**Table 1:** Relative abundance (%) of families in cecal digesta of broiler chickens fed diets with increasing levels of deoxynivalenol (DON; 0, 2.5, 5, or 10 mg DON/kg diet) and with or without oral lipopolysaccharide challenge (LPS) 1 day prior slaughter^1^.

	**no LPS**				**LPS**					**Fixed effect**, ***p*****-values**			**Contrasts**, ***p*****-values^2^**		
**DON (mg/kg feed)**	**0**	**2.5**	**5**	**10**	**0**	**2.5**	**5**	**10**	**SEM**	**DON**	**LPS**	**DON × LPS**	**0 vs. DON**	**lin**.	**quad**.
*Clostridiales* family 1	34.99	39.00	31.83	47.66	29.74	37.48	39.09	45.33	8.39	0.38	0.94	0.89	0.26	0.14	0.66
*Ruminococcaceae*	31.12	32.97	31.30	30.09	34.06	28.87	25.32	30.85	4.55	0.82	0.62	0.74	0.47	0.54	0.55
*Enterobacteriaceae*	15.44	12.49	12.43	8.88	14.61	15.44	17.36	8.63	3.06	0.14	0.43	0.76	0.32	0.07	0.24
*Turicibacteraceae*	9.51	7.09	10.90	5.68	11.58	9.61	8.75	6.93	3.75	0.69	0.73	0.92	0.44	0.35	0.80
*Lachnospiraceae*	5.71	6.84	10.88	6.03	7.57	6.58	7.07	5.59	1.78	0.33	0.60	0.46	0.72	0.97	0.20
*Lactobacillaceae*	0.58	0.45	0.47	0.37	0.44	0.90	0.60	0.28	0.22	0.48	0.59	0.55	0.97	0.34	0.23
kingdom unassigned	0.61	0.30	0.30	0.35	0.33	0.39	0.32	0.37	0.07	0.13	0.46	0.06	0.02	0.11	0.08
*Clostridiaceae*	0.47	0.14	0.25	0.14	0.32	0.11	0.07	0.14	0.08	<0.01	0.12	0.66	<0.01	0.01	0.03
*Clostridiales* family 2	0.15	0.18	0.15	0.20	0.11	0.12	0.12	0.17	0.02	0.08	0.03	0.97	0.14	0.04	0.38
*Coriobacteriaceae*	0.11	0.10	0.07	0.14	0.10	0.21	0.09	0.15	0.05	0.31	0.33	0.54	0.58	0.80	0.83
*Erysipelotrichaceae*	0.13	0.06	0.06	0.07	0.11	0.11	0.08	0.15	0.04	0.60	0.32	0.67	0.34	0.72	0.20
*Corynebacteriaceae*	0.00	0.28	0.06	0.10	0.11	0.03	0.03	0.03	0.08	0.52	0.26	0.17	0.60	0.79	0.51
*Enterococcaceae*	0.02	0.04	0.05	0.01	0.02	0.02	0.08	0.02	0.02	0.14	0.74	0.79	0.42	0.77	0.09
*Peptostreptococcaceae*	0.02	0.02	0.02	0.01	0.03	0.03	0.04	0.01	0.01	0.14	0.16	0.69	0.46	0.29	0.25

1 *Data are presented as least squares means ± standard error of the mean (SEM); n = 10 per treatment group*.

2 *P-values for orthogonal contrasts to test linear (lin.) and quadratic (quad.) relationships between control feeding and the 3 increasing levels of DON as well as the overall difference of 0 DON vs. all DON groups (0 vs. DON)*.

**Table 2:** Differences in relative abundance (%) of genera in cecal digesta of broiler chickens fed diets with increasing levels of deoxynivalenol (DON; 0, 2.5, 5, or 10 mg DON/kg diet) and with or without oral lipopolysaccharide challenge (LPS) 1 day prior slaughter^1^.

	**no LPS**				**LPS**					**Fixed effect**, ***p*****-values**			**Contrasts**, ***p*****-values^2^**		
**DON (mg/kg feed)**	**0**	**2.5**	**5**	**10**	**0**	**2.5**	**5**	**10**	**SEM**	**DON**	**LPS**	**DON × LPS**	**0 vs. DON**	**lin**.	**quad**.
*Enterobacteriaceae* genus 1	15.24	11.94	10.80	8.14	14.18	14.41	16.52	8.51	2.84	0.12	0.35	0.66	0.20	0.04	0.35
*Oscillospira*	3.16	1.81	2.84	2.09	2.71	1.59	2.23	2.74	0.35	0.01	0.52	0.28	0.01	0.51	0.03
*Anaerotruncus*	1.04	1.32	2.51	1.44	1.47	1.44	2.80	1.32	0.51	0.02	0.62	0.95	0.19	0.31	0.05
*Blautia*	0.33	0.76	1.23	0.54	0.54	0.99	0.86	0.72	0.21	0.03	0.69	0.43	0.02	0.26	0.01
unassigned bacterial phylum	0.61	0.30	0.30	0.35	0.33	0.39	0.32	0.37	0.07	0.13	0.46	0.06	0.02	0.11	0.08
*Clostridiales* genus 2	0.15	0.18	0.15	0.20	0.11	0.12	0.12	0.17	0.02	0.08	0.03	0.97	0.14	0.04	0.38
*Clostridiaceae* genus	0.28	0.06	0.14	0.08	0.13	0.04	0.02	0.07	0.08	0.18	0.17	0.73	0.03	0.15	0.16
*Clostridium*	0.20	0.08	0.10	0.06	0.19	0.07	0.05	0.06	0.03	<0.01	0.42	0.78	<0.01	<0.01	0.01
*Dorea*	0.07^b^	0.08^b^	0.18^a^	0.08^b^	0.14^ab^	0.11^ab^	0.08^b^	0.07^b^	0.03	0.15	0.94	0.01	0.81	0.42	0.16
*Enterobacteriacee* genus 2	0.015	0.013	0.016	0.036	0.010	0.016	0.017	0.019	0.007	0.14	0.38	0.50	0.22	0.04	0.36
*Ruminococcaceae* genus 2	0.010	0.005	0.003	0.003	0.008	0.006	0.002	0.004	0.002	0.05	0.87	0.84	0.01	0.02	0.15

1 *Data are presented as least squares means ± standard error of the mean (SEM); n = 10 per treatment group; only values for genera that were different (p < 0.05) are presented*.

2 *P-values for orthogonal contrasts to test linear (lin.) and quadratic (quad.) relationships between control feeding and the 3 increasing levels of DON as well as the overall difference of 0 DON vs. all DON groups (0 vs. DON)*.

a, b *DON × LPS interaction: Least squares means of genera with no common superscripts differ significantly between groups; p < 0.05*.

### Effects of DON and LPS treatment on bacterial composition

**Figure 2:**
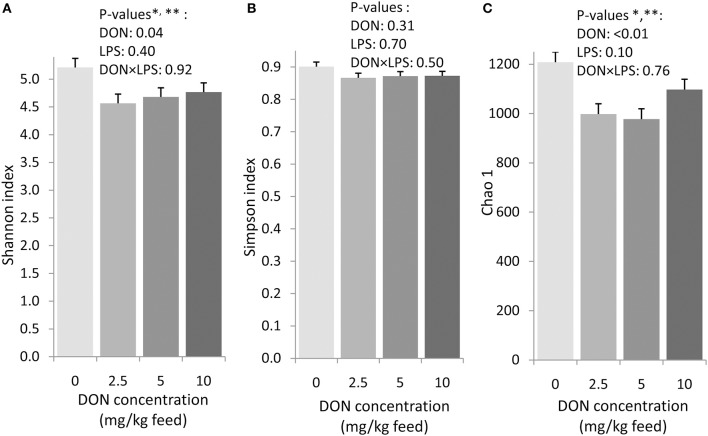
Shannon index **(A)**, Simpson index **(B)**, and Chao1 richness estimate **(C)** in cecal digesta of chickens fed diets with increasing levels of deoxynivalenol (DON; 0, 2.5, 5, or 10 mg DON/kg diet). Values are presented as least squares means ± standard error of the mean (SEM); *n* = 20 broilers per feeding group. Presented data include both animals with and without oral lipopolysaccharide challenge (LPS) 1 day prior to slaughter within the respective feeding group. ^*^ Contrast comparing the 0 DON with all DON groups (0 vs. DON): *p* < 0.05. ^**^ Quadratic contrast: *p* < 0.05.

The exposure to increasing levels of DON decreased (*p* < 0.05) the cecal species richness (Chao1) and evenness (Shannon) by up to 0.2- and 0.1-fold, respectively, compared to 0 DON (Figure 2). Increasing DON levels also affected the bacterial community composition as high as the phylum level. The greatest alterations were observed within the predominant phyla *Firmicutes* and *Proteobacteria* which tended (*p* < 0.10) to linearly increase and decrease by up to 0.1- and 0.4-fold with increasing DON-levels, respectively (Figure 1). Accordingly, the ratio of *Firmicutes* to *Proteobacteria* tended to linearly increase (*p* = 0.08) by up to 0.9-fold with increasing DON-concentrations (Supplementary Table S2). The most pronounced DON-induced changes at family level were a 0.6-fold decrease in the relative abundance of *Clostridiaceae* in the DON groups (*p* < 0.01) and a linear increase in an unassigned *Clostridiales* family 2 by 0.5-fold with increasing DON concentrations (*p* = 0.04). This was reflected at genus level where *Oscillospira, Clostridiaceae genus, Clostridium*, and the unassigned *Ruminoccaceae* genus 2 were decreased by the DON treatment (*p* < 0.05). *Oscillospira* and the unassigned *Ruminoccaceae* genus 2 decreased by 0.2- and 0.5-fold in the DON groups compared to the 0 DON group, respectively, whereas *Clostridium* linearly decreased by up to 0.7-fold with increasing DON concentrations. DON further quadratically increased the relative abundance of *Blautia* (*p* = 0.01) and *Anaerotruncus* (*p* = 0.05) by up to 1.4- and 1.1-fold with the highest abundance in the 5 DON group. Moreover, an unassigned *Enterobacteriaceae* genus 1 linearly decreased (*p* < 0.05) with increasing DON-concentrations by up to 0.4-fold. Within these genera, the DON treatment affected 38 of the 120 most abundant OTUs (relative abundance > 0.05; Supplementary Figure S1). Among those, 5 OTUs could be assigned to >95% similarity to reference strains (Supplementary Table S3). Increasing DON levels linearly decreased *Escherichia/Shigella*-OTU2 by up to 0.5-fold and two *Salmonella*-OTUs (OTU 70 and 73) by up to 0.7- and 0.5-fold, respectively (*p* < 0.05). Two *Anaerotruncus*-OTUs (OTU 18 and 22) tended to be quadratically affected by the increasing DON levels, reaching their highest abundance in cecal digesta of chickens in the 5 DON group.

The oral LPS challenge decreased the relative abundance of an unassigned *Clostridiales* genus 2 by 0.2-fold compared to the negative control (*p* = 0.03). At OTU level, the relative abundance of only 4 out of the 120 most abundant OTUs were modified by the LPS treatment.

Moreover, the DON × LPS interaction (*p* = 0.01) for the genus *Dorea* indicated that there was a quadratic response of *Dorea* to the increasing DON concentrations in chickens that did not receive the LPS challenge. In chickens that were additionally challenged with LPS increasing DON levels did not modify the relative abundance of *Dorea*.

### Effects of DON and LPS treatment on short-chain fatty acids

**Figure 3:**
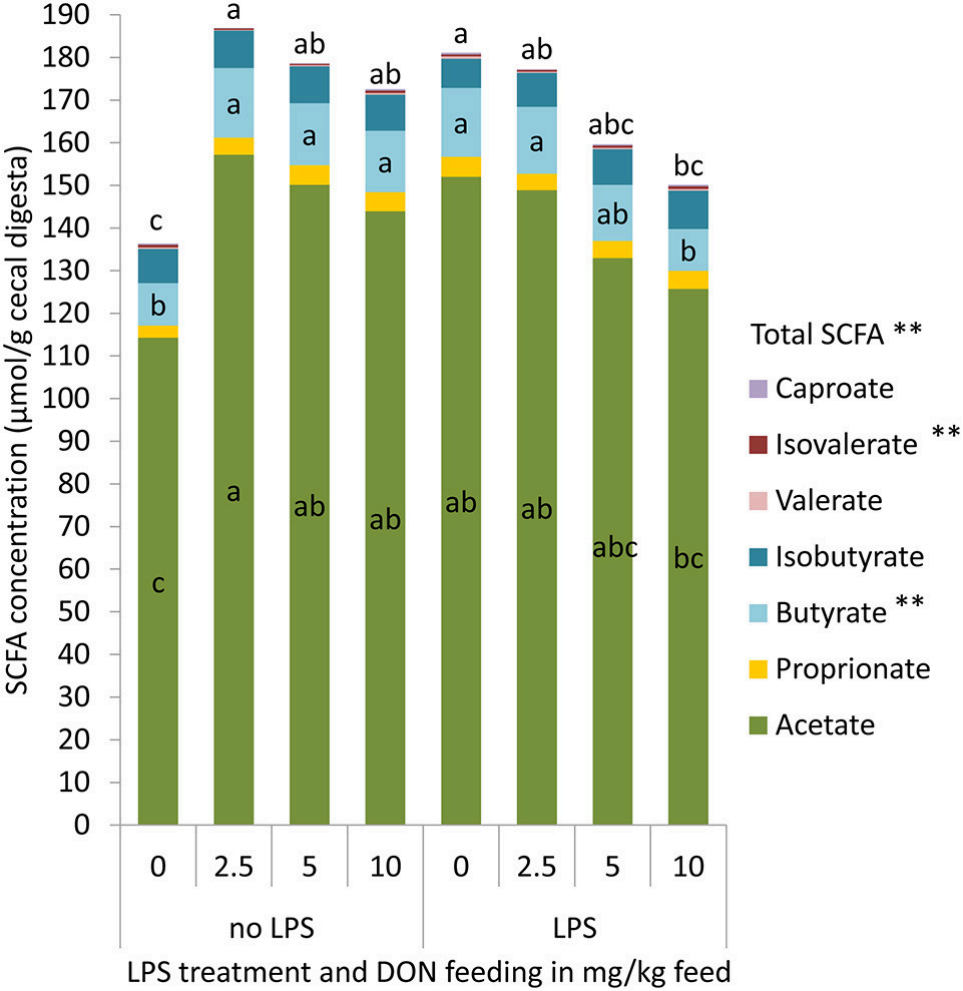
Total short chain fatty acids (SCFA), acetate, proprionate, butyrate, isobutyrate, valerate, isovalerate, and caproate concentrations in cecal digesta of chickens fed diets with increasing levels of deoxynivalenol (DON; 0, 2.5, 5, or 10 mg DON/kg diet) and with or without oral lipopolysaccharide challenge (LPS) 1 day prior to slaughter. Values are presented as least squares means ± standard error of the mean (SEM); *n* = 10 broilers per treatment; *n* = 9 in the 5 DON+con and 5 DON+lps groups. ^a,b,c^ DON × LPS interaction: Least squares means of total or individual SCFA with no common superscripts differ significantly between groups; *p* < 0.05. ^**^Quadratic contrast: *p* < 0.05.

Increasing dietary concentrations of DON quadratically affected the cecal total SCFA and butyrate concentration (*p* < 0.05, Figure 3), with the highest concentration in the 2.5 DON group (Figure 3). The DON × LPS interactions for total SCFA (*p* = 0.01) and butyric acid (*p* < 0.01) further indicated that those effects were only observed in chickens that were not challenged with LPS. In chickens that received the LPS, SCFA concentrations linearly decreased with increasing DON concentrations. Furthermore, an opposite quadratic relationship between iso-valerate and increasing DON concentrations was observed (*p* = 0.01), with the lowest concentration of iso-valerate being found in the 2.5 and 5 DON groups.

### Effect of DON and LPS treatments on functional metagenome prediction

Functional metagenome prediction was used to assess potential alterations in bacterial metabolism caused by the dietary DON levels (Table 3; Supplementary Table S4). The relative gene abundance of COG pathways for metabolism of co-factors and vitamins, cell motility, and biosynthesis of other secondary metabolites were linearly increased with increasing dietary DON levels (*p* < 0.05; Table 3). The relative abundance of COG pathway genes for lipid metabolism, genetic information processing and glycan biosynthesis and metabolism, in turn, were linearly decreased by increasing DON levels (*p* < 0.05). In total, 35 KEGG pathways were less abundant in chickens fed the DON diets compared to the 0 DON diet, including KEGG pathways for amino acid, carbohydrate, and lipid metabolism and cell replication. In contrast, 21 KEGG pathways were more abundant in chickens fed the DON diets compared to those fed the 0 DON diet and were related to bacterial motility, cell metabolism, amino acid, vitamin, and antimicrobial metabolism. The LPS treatment, in turn, did not affect the relative abundance of predicted metabolic pathways.

**Table 3:** Differences in relative abundance (%) of COG pathways (Cluster of orthologous groups of proteins) in cecal digesta of broiler chickens fed diets with increasing levels of deoxynivalenol (DON; 0, 2.5, 5, or 10 mg DON/kg diet) and with or without oral lipopolysaccharide challenge (LPS) 1 day prior slaughter^1^.

	**no LPS**				**LPS**					**Fixed effect**, ***p*****-values**			**Contrasts**, ***p*****-values^2^**		
**DON (mg/kg feed)**	**0**	**2.5**	**5**	**10**	**0**	**2.5**	**5**	**10**	**SEM**	**DON**	**LPS**	**DON × LPS**	**0 vs. DON**	**lin**.	**quad**.
**CELLULAR PROCESSES**															
Cell motility	2.94	3.47	3.20	3.68	2.98	3.42	3.44	3.67	0.27	0.08	0.77	0.95	0.02	0.02	0.73
Transport and catabolism	0.18	0.17	0.17	0.17	0.18	0.17	0.16	0.17	0.01	0.10	0.70	0.67	0.02	0.05	0.13
**METABOLISM**															
Metabolism of cofactors and vitamins	3.87	4.05	4.00	4.08	3.92	4.02	4.01	4.05	0.07	0.08	0.96	0.94	0.01	0.03	0.41
Lipid metabolism	2.92	2.87	2.89	2.82	2.93	2.86	2.85	2.81	0.04	0.12	0.73	0.97	0.05	0.03	0.96
Enzyme families	2.13	2.11	2.12	2.11	2.14	2.10	2.10	2.11	0.01	0.13	0.54	0.70	0.02	0.09	0.12
Glycan biosynthesis and metabolism	1.68	1.62	1.61	1.58	1.68	1.67	1.65	1.59	0.04	0.16	0.40	0.92	0.09	0.03	0.84
Metabolism of terpenoids and polyketides	1.58	1.53	1.54	1.51	1.55	1.53	1.53	1.51	0.02	0.05	0.58	0.83	0.01	0.02	0.60
Biosynthesis of other secondary metabolites	0.74	0.79	0.79	0.79	0.77	0.78	0.77	0.79	0.01	<0.01	0.88	0.09	<0.01	<0.01	0.37
**UNCLASSIFIED**															
Poorly characterized	5.00	4.84	4.88	4.79	4.97	4.93	4.92	4.82	0.07	0.10	0.51	0.88	0.04	0.02	0.93
Genetic information processing	2.78^a^	2.70^bc^	2.69^b^	2.71^bc^	2.73^abc^	2.76^ac^	2.73^abc^	2.71^bc^	0.02	0.08	0.43	0.04	0.02	0.02	0.37

1 *Data are presented as least squares means ± standard error of the mean (SEM); n = 10 per treatment group; only values for COG pathways that were different (p < 0.05) are presented*.

2 *P-values for orthogonal contrasts to test linear (lin.) and quadratic (quad.) relationships between control feeding and the three increasing levels of DON as well as the overall difference of 0 DON vs. all DON groups (0 vs. DON)*.

a, b, c *DON × LPS interaction: Least squares means of COG pathways with no common superscripts differ significantly between groups; p < 0.05*.

### Correlation analysis

**Figure 4:**
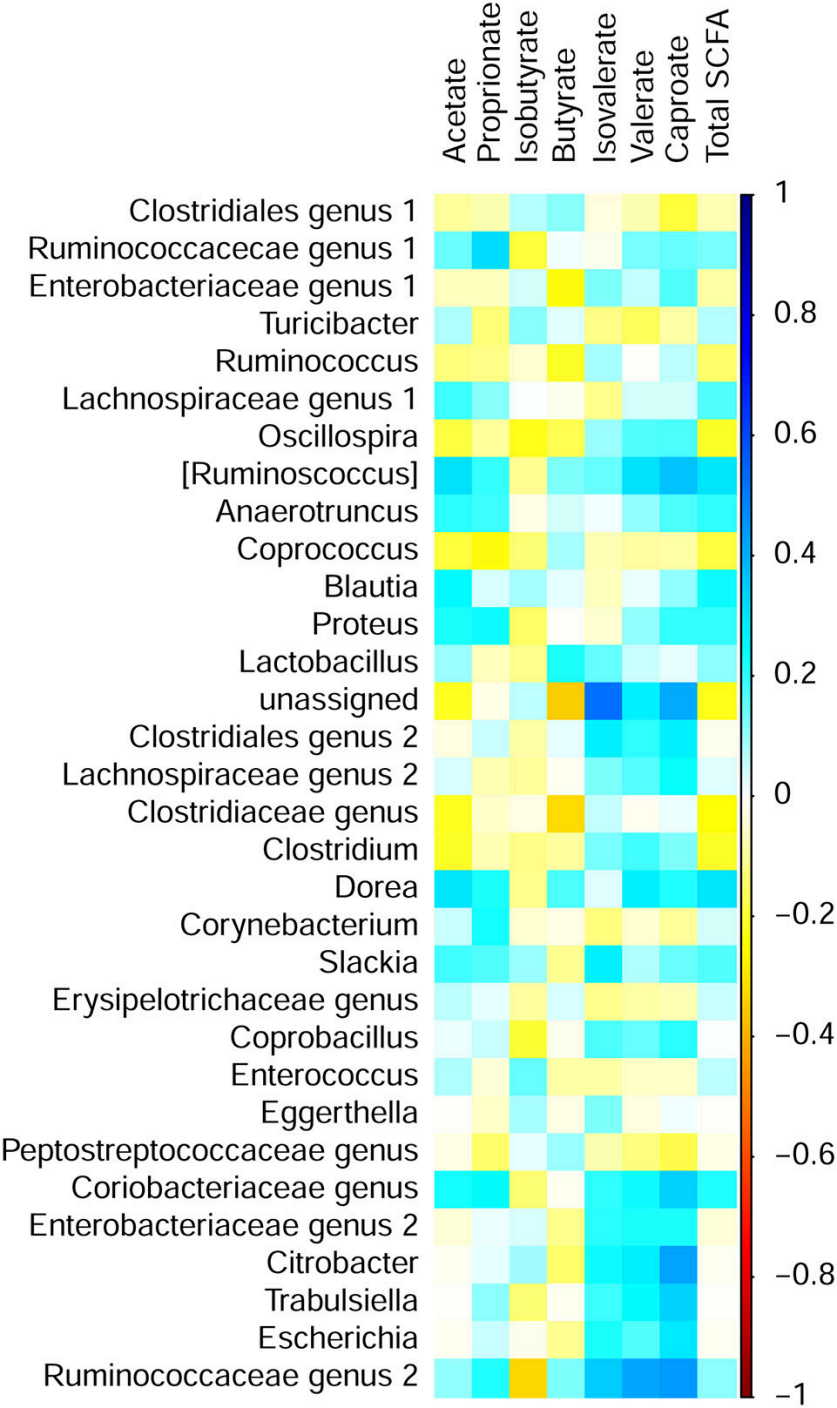
Correlation matrix between short chain fatty acid (SCFA) concentrations and relative abundance of bacterial genera in cecal digesta of chickens fed diets with increasing levels of deoxynivalenol (DON; 0, 2.5, 5, or 10 mg DON/kg diet) and with or without oral lipopolysaccharide challenge (LPS) 1 day prior to slaughter. Colors refer to the degree of correlation.

Pearson's correlation analysis was used to characterize associations of bacterial abundances with SCFA concentrations and predicted metabolic functions in cecal digesta. In total, 15 significant correlations between bacterial genera and SCFA could be established (Figure 4). Most correlations were observed within the phylum *Firmicutes*. *Ruminococcaceae* genus 2 positively correlated with valerate (*r* = 0.42). Moreover, several genera positively correlated with caproate including [*Ruminococcus*] (*r* = 0.36), *Citrobacter* (*r* = 0.42), and *Ruminococcaceae* genus 2 (*r* = 0.44). Furthermore, a negative correlation between body weight at the day of sampling (Lucke et al., 2017a) and caproate (*r* = −0.37) could be established. In addition, slaughter weight positively correlated with *Clostridiales* genus 1 (*r* = 0.38). However, correlations of slaughter weight with *Blautia* (*r* = −0.36) and *Escherichia* (*r* = −0.36) were negative.

Correlations between the abundances of bacterial genera and COG or KEGG pathways were detected for the five most abundant genera and many low abundant genera (Supplementary Figures S1, S2). Especially the high abundant *Clostridiales* genus 1 and *Enterobacteriaceae* genus 1 showed opposite relationships with several COG pathways. *Clostridiales* genus 1 was positively correlated with COG pathways for translation and transcription (*r* > 0.70). *Enterobacteriaceae* genus 1, however, was (*r* < −0.70) negatively correlated with the aforementioned COG pathways. By contrast, *Clostridiales* genus 1 negatively correlated (*r* < −0.70) with COG pathways for cellular processes and signaling, metabolism and glycan biosynthesis and metabolism, whereas *Enterobacteriaceae* genus 1 positively correlated with these pathways. A comparable pattern was observed for correlations between genera and respective KEGG pathways. *Clostridiales* genus 1 was further highly negatively correlated with the COG pathway for lipid metabolism (*r* = −0.94). Moreover, positive correlations were also detected for an *Enterobacteriaceae* genus and glycan biosynthesis and metabolism (*r* = 0.92) as well as between *Turicibacter* and the COG pathway for signaling molecules and interaction (*r* = 0.91).

## Discussion

The present results demonstrated that different levels of dietary DON contamination have the potential to alter the cecal bacterial diversity and composition in chickens, which was especially evident within the two predominant phyla *Firmicutes* and *Proteobacteria*, modifying the *Firmicutes*-to-Proteobacteria ratio toward more *Firmicutes* bacteria. DON-related changes in predicted bacterial metagenomic functions and cecal SCFA concentrations reflected the alterations in the bacterial community composition. Notably, unclassified members of the order *Clostridiales* benefited from the increasing dietary DON levels, which may have benefited from the opening intestinal niche due to the DON-related decline in the *Enterobacteriaceae* population. Nevertheless, the decreased bacterial richness and diversity suggested that certain taxa within the bacterial community may have had growth advantages due to the DON exposure which may have lowered the total number and abundance of species. There is some debate whether a reduced bacterial diversity is detrimental for the host or not. Previously, it was thought that high species diversity may reflect a more stable microbiota by preventing the colonization of pathogens (Han et al., 2017). However, a less diverse, but more specialized bacterial community was proposed to use limiting resources more efficiently and promote the energy acquisition of the host (Lozupone et al., 2012; Siegerstetter et al., 2017). Chickens were fed the contaminated diets from the first day of life. Therefore, it is conceivable that DON may have modified the initial bacterial colonization of chicken's gut. Also, chickens receiving the higher DON contamination levels reduced their feed intake (Lucke et al., 2017a), which may partly explain the quadratic effects of dietary DON on the cecal microbiota composition and α-diversity in the present study. The additional oral challenge with a highly immunogenic *E. coli*-LPS only little modulated the DON effects on the bacterial community. However, the LPS challenge appeared to enhance microbial activity, as indicated by the cecal SCFA concentrations, but only in chickens receiving the non-contaminated diet. This finding may have been related to LPS effects on mucin secretion as well as on digestion and absorption in the small intestine and consequently intestinal nutrient flow (Smirnova et al., 2003; Amador et al., 2007; Mani et al., 2012; Zhang et al., 2017).

Because DON largely impacts the feed intake (Lucke et al., 2017a) as well as digestive, absorptive and immune functions in the small intestine of chickens (Awad et al., 2011b; Ghareeb et al., 2013; Osselaere et al., 2013), DON-effects on the cecal bacterial microbiota were probably rather indirect effects due to changes in cecal nutrient flow and mucus secretion (Antonissen et al., 2015). In line with that, only about 20% of the dietary DON were reported to reach the chicken ceca (Awad et al., 2011a) due to mucosal absorption, sulfation and bacterial de-epoxidization (Gratz et al., 2013; Schwartz-Zimmermann et al., 2015). Bacterial isolates from the chicken intestine, being able to transform DON to deepoxy-deoxynivalenol (DOM-1), were reported to belong to *Clostridiales, Anaerofilum, Collinsella*, and *Bacillus* (Yu et al., 2010). In the present study, the dietary exposure to DON specifically promoted the relative abundance of an unclassified *Clostridiales* genus. This genus was comprised by OTUs which showed 83.4% sequence similarity to proteolytic species within *Clostridium* sensu strictu. However, due to the high versatility within the order *Clostridiales*, the low taxonomic resolution at species level rendered it difficult to deduce specific metabolic abilities; particularly, as many members within *Clostridiales* have been poorly described and the need to re-annotate many *Clostridium* species within the 16S rRNA gene tree (Stackebrandt et al., 1999; Biddle et al., 2013). Therefore, we can only speculate that changes in the cecal mucin expression or increased protein flow to the ceca may explain the increased abundance of these taxa. In this context, results of DON-effects on the branched-chain fatty acids were less conclusive to indicate whether more or less substrate was available in the cecal lumen for bacterial protein fermentation. Nevertheless, due to the low similarities to cultured strains and the DON-related decrease in the genus *Clostridium*, it remains speculative which species were affected and whether this increase was related to DON-degrading capabilities, changes in cecal physiology or competition for intestinal niches with other bacteria. Concurrently, we observed a decrease in the relative abundance of the predominant *Enterobacteriaceae* genus and within it *Escherichia/Shigella*-OTU2, which is adept at utilizing host mucus as substrate (Zhu and Joerger, 2003; Tenaillon et al., 2010; Conway and Cohen, 2015). Lower cecal mucin production as cause for the reduced *Enterobacteriaceae* abundance with increasing DON levels was supported by the positive correlation between the unclassified *Enterobacteriaceae* genus and the COG pathway of glycan biosynthesis and metabolism as well as by the lower abundances of metabolic pathways for glycosaminoglycan degradation and amino acid metabolism after DON exposure. As *Enterobacteriaceae*, such as *E. coli*, belong to the commensal intestinal microbiota in chickens but also comprise pathobionts (Dozois et al., 2003; Smati et al., 2015), it is difficult to deduce whether this decrease was beneficial for or disturbed the cecal bacterial homeostasis. By contrast, the lower relative abundance of *Proteobacteria*, in general, and more specifically of *Enterobacteriaceae* with increasing dietary DON levels may have lowered the cecal mucosal exposure to LPS of high immune reactivity (Gronbach et al., 2014), as indicated by the lower abundance of the KEGG pathway of LPS biosynthesis proteins. This may have reduced the LPS-related mucosal innate immune response (Yang and Jobin, 2014). DON-related alterations within the *Ruminococcaceae* and *Lachnospiraceae* may be due to DON-related changes in intestinal nutrient flow and availability in chickens (Awad et al., 2011b). Moreover, correlation analysis with SCFA and the relative abundance of the KEGG pathway of fatty acid biosynthesis indicated that members of the families *Ruminococcaceae* and *Lachnospiraceae* (Flint et al., 2015; Polansky et al., 2015) may have been associated with the observed DON-related changes in cecal SCFA concentrations. In this regard, *Ruminococcaceae* and *Lachnospiraceae*, including *Blautia, Dorea*, and *Ruminococcus*, are known for their capability to degrade cellulose and hemicelluloses (Biddle et al., 2013). DON may have affected the cross-feeding of primary fermentation metabolites, such as lactate or succinate (Flint et al., 2015), or metabolism of gases among microbes in cecal digesta (Rajilić-Stojanović and De Vos, 2014), which may have modified *Ruminococcaceae* and *Lachnospiraceae* abundances as well. Corresponding to our findings, *Oscillospira*, a predominant genus in chicken cecal microbiota (Wang et al., 2016) has been previously found to decrease in inflammatory diseases in humans (Zhu et al., 2013; Walters et al., 2014; Gophna et al., 2017), whereas *Anaerotruncus* was positively associated with intestinal inflammatory diseases, such as colorectal cancer (Chen et al., 2012).

Due to its lipid A moiety linked to an antigenic O-polysaccharide (Heinrichs et al., 1998), the currently used LPS from *E. coli* O55:B5 was a highly immunogenic antigen capable to induce an intestinal inflammatory response after repeated oral application of 250 μg/kg BW in chickens (Wu et al., 2013). The related mucosal immune response may have therefore potentiated the effects of increasing DON levels on gut physiology (e.g., mucin expression) and digestive processes with consequences for microbial colonization in the present study. The latter assumption may explain the higher relative abundance of *Clostridiales*-OTU24 and the decrease in *Ruminococcaceae*-OTU67 and OTU93 after the LPS administration across all DON levels. However, the strongest effect of LPS was found for *Clostridiales*-OTU12 and SCFA concentrations, mainly acetate and butyrate, but only in chickens receiving the 0 DON diet. This may indicate that DON and LPS exposure (e.g., nutrient flow and mucin secretion) may have caused similar environmental conditions for bacterial proliferation and activity in cecal digesta.

In conclusion, the present results demonstrated that the different DON contamination levels of chicken feed substantially modulated the cecal bacterial microbiota composition and decreased the bacterial diversity. DON-related alterations in the bacterial community were especially evident by an increase in an unclassified *Clostridiales* genus and a decrease in the family *Enterobacteriaceae*. Changes in host digestive physiology and mucin expression as reason for the altered intestinal bacterial abundances were indicated by DON-related alterations in KEGG pathway abundance genes related to glycoprotein and amino acid metabolism and increased cecal SCFA concentrations. Further studies are needed to clarify which DON-related changes in the cecal microbiota and SCFA were caused by bacterial DON degradation and those which were due to DON-induced changes in host physiology. The additional oral challenge with a highly immunogenic *E. coli*-LPS showed the strongest effect in chickens that received the diet without DON contamination and mainly responded with an increased cecal SCFA concentration.

## Author contributions

JB and QZ conceived and designed the experiments and revised the manuscript; AL performed the experiments; AL and BM-Z analyzed the data, interpreted the data, and drafted the manuscript. All of the authors read and approved the final manuscript.

### Conflict of interest statement

The authors declare that the research was conducted in the absence of any commercial or financial relationships that could be construed as a potential conflict of interest.
