# Supplementary material for: Dietary Deoxynivalenol Contamination and Oral Lipopolysaccharide Challenge Alters the Cecal Microbiota of Broiler Chickens
Source: Front Microbiol. 2018 Apr 25;9:804. doi: 10.3389/fmicb.2018.00804 (PMC5996912; doi:10.3389/fmicb.2018.00804)
Supplement: Supplementary file 1 [file Data_Sheet_1.PDF]

Supplementary Material

Feeding Low to Moderate Doses of Deoxynivalenol and Oral Lipopolysaccharide Challenge alters the Cecal Microbiota of Broiler Chickens

Annegret Lucke, Josef Böhm, Qendrim Zebeli, Barbara U. Metzler-Zebeli*

*** Correspondence:** Barbara U. Metzler-Zebeli, Barbara.Metzler@vetmeduni.ac.at

1 Supplementary Figures and Tables

1.1 Supplementary Figures

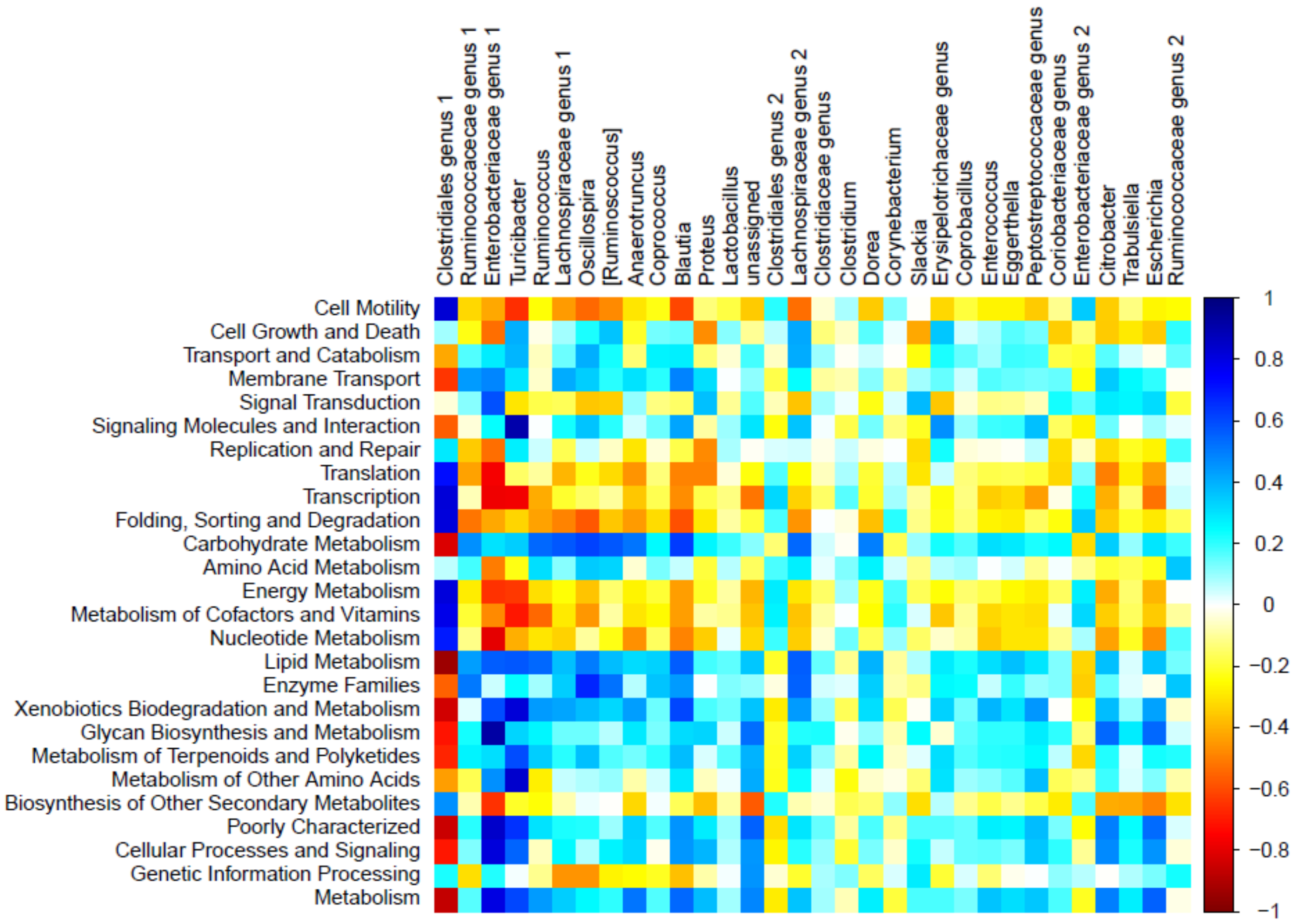

Supplementary Figure S1. Correlation matrix between Cluster of Orthologous Groups of proteins (COG) pathways and bacterial genera in cecal digesta of chickens fed diets with increasing levels of deoxynivalenol (DON; 0, 2.5, 5 or 10 mg DON/kg diet) and with or without oral lipopolysaccharide challenge (LPS) 1 day prior to slaughter.

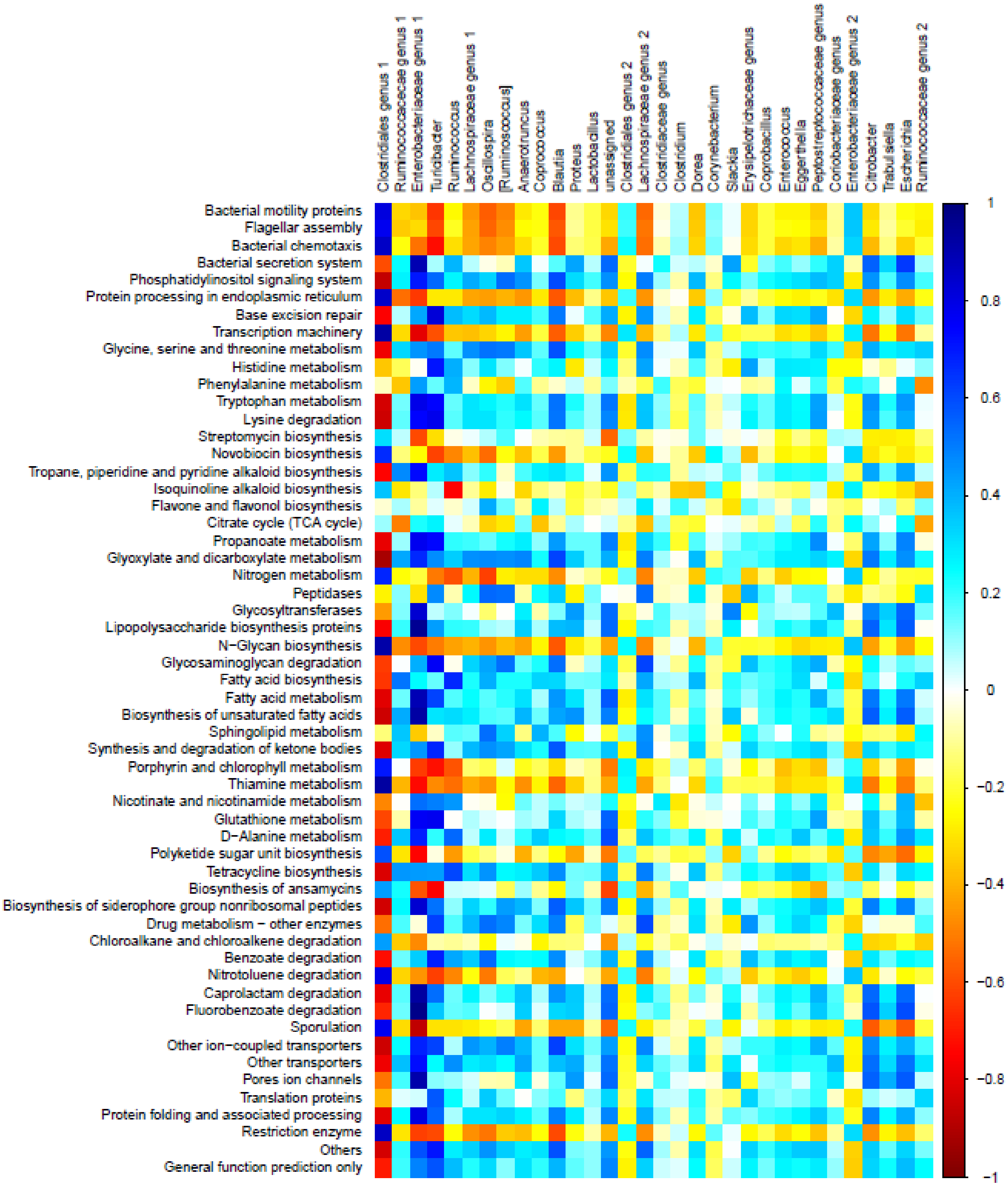

Supplementary Figure S2. Correlation matrix between Kyoto Encyclopedia of Genes and Genomes (KEGG) pathways and bacterial genera in cecal digesta of chickens fed diets with increasing levels of deoxynivalenol (DON; 0, 2.5, 5 or 10 mg DON/kg diet) and with or without oral lipopolysaccharide challenge (LPS) 1 day prior to slaughter. Only KEGG pathways that differed significantly between treatments ($p < 0.05$) were correlated.

1.2 Supplementary Tables

Supplementary Table S1. Ingredients and nutrient composition of the basal diet which was used to mix the DON-contaminated diets (Lucke et al., 2017a).

Ingredients (kg per 100 kg feed)	
Wheat [11% crude protein]	57.59
Soy bean meal	25.44
Supplement[1,2]	13.00
Rapeseed oil	2.97
Megafat	1.00
**Analytical composition (g per kg fresh matter):**	
Dry matter	889
Crude protein	207
Crude fat	78
Crude fiber	30
Crude ash	73
Starch	365
Sugar	52
Calculated metabolizable energy (MJ/kg)	12.66

[1]Composition of the supplement: soybean, toasted 46.2%; calcium carbonate 13%; monocalcium phosphate 12.6% hardened palm kernel oil 12.5%; pumpkin seed cake 4.8%; sodium bicarbonate 2.3%; sodium chloride 1.5%; magnesium phosphate 0.9%.

[2]per kg product: dry matter 94%; calcium 7.2%; phosphorus 3.3%; sodium 1.2%; iron 850 mg; copper 160 mg; zinc 500 mg; manganese 500 mg; iodine 16.6 mg; selenium 3 mg; Vitamin A 90000 IE; Vitamin D3 33300 IE; Vitamin E 650 mg; Vitamin K3 25 mg; Vitamin B1 25 mg; Vitamin B2 60 mg; Vitamin B6 50 mg; Vitamin B12 300 μg; pantothenic acid 115 mg; nicotinic acid 610 mg; vitamin C 600 mg; folic acid 16 mg; biotin 1800 μg; choline chloride 4700 mg; lysine 2.9%; methionine 1.9%; threonine 1.5%; butylated hydroxytoluene (E321) 1000 mg.

Supplementary Table S2. Relative abundance (%) of operational taxonomic units (OTU) and ratio of *Firmicutes*/*Proteobacteria* in cecal digesta of broiler chickens fed diets with increasing levels of deoxynivalenol (DON) and with or without oral lipopolysaccharide challenge (LPS) 1 day prior to slaughter.[1]

	no LPS				LPS					Fixed effect, *p*-values			Contrasts, *p*-values[1]			
DON (mg/kg feed)	0	2.5	5	10	0	2.5	5	10	SEM	DON	LPS	DON × LPS	0 vs. DON	lin.	quad.	Taxonomy
OTU1	5.04	16.77	13.37	18.80	5.79	15.75	15.46	18.61	5.23	<0.01	0.85	0.96	<0.01	<0.01	0.14	*Clostridiales* (o)
OTU2	12.45	10.02	8.65	6.39	11.95	12.24	14.08	6.99	2.47	0.12	0.27	0.65	0.22	0.04	0.31	*Enterobacteriaceae* (f)
OTU3	2.52	8.57	6.85	9.46	3.16	8.21	7.99	9.49	2.66	0.07	0.85	0.99	0.01	0.03	0.36	*Clostridiales* (o)
OTU11	0.77	2.42	1.96	2.70	0.97	2.34	2.29	2.57	0.74	0.09	0.88	0.99	0.02	0.04	0.35	*Clostridiales* (o)
OTU12	12.88[a]	0.10[b]	0.00[b]	0.36[b]	0.01[b]	0.02[b]	0.36[b]	0.05[b]	2.07	0.01	0.03	<0.01	<0.01	0.01	0.03	*Clostridiales* (o)
OTU13	1.54	1.45	1.10	1.56	2.50	1.00	0.95	2.01	0.45	0.10	0.53	0.41	0.07	0.53	0.02	*Clostridiales* (o)
OTU15	0.59	1.20	1.40	0.77	0.84	1.11	1.42	0.76	0.36	0.17	0.87	0.97	0.18	0.72	0.04	*Lachnospiraceae* (f)
OTU16	1.07	0.72	0.76	0.83	1.34	1.31	0.77	0.65	0.25	0.21	0.33	0.46	0.08	0.04	0.66	*Ruminococcus* (g)
OTU18	0.46	0.65	1.26	0.72	0.72	0.72	1.37	0.64	0.25	0.02	0.62	0.93	0.15	0.27	0.05	*Anaerotruncus* (g)
OTU22	0.41	0.52	1.01	0.57	0.61	0.58	1.16	0.54	0.21	0.02	0.53	0.94	0.21	0.33	0.06	*Anaerotruncus* (g)
OTU24	0.40	0.38	0.41	0.53	0.83	0.67	0.98	0.66	0.25	0.93	0.05	0.84	0.96	0.89	0.98	*Clostridiales* (o)
OTU28	0.85	0.30	0.24	0.55	0.68	0.32	0.38	0.45	0.17	0.03	0.84	0.81	0.01	0.15	0.01	*Ruminococcaceae* (f)
OTU29	0.44	0.50	0.30	0.31	0.56	0.39	0.25	0.41	0.09	0.07	0.79	0.53	0.06	0.04	0.26	*Ruminococcaceae* (f)
OTU41	0.05	0.38	0.57	0.19	0.09	0.32	0.42	0.22	0.14	0.02	0.74	0.91	0.02	0.23	<0.01	*Blautia producta* (s)
OTU42	0.34	0.20	0.27	0.18	0.33	0.18	0.30	0.26	0.06	0.05	0.66	0.81	0.02	0.15	0.34	*Oscillospira* (g)
OTU43	0.20	0.17	0.65	0.21	0.25	0.18	0.24	0.20	0.10	0.04	0.19	0.09	0.55	0.51	0.20	*Lachnospiraceae* (f)
OTU47	0.39	0.10	0.25	0.19	0.33	0.07	0.23	0.22	0.08	0.01	0.72	0.96	0.01	0.21	0.04	*Oscillospira* (g)
OTU48	0.28	0.07	0.22	0.32	0.32	0.21	0.13	0.28	0.09	0.18	0.82	0.63	0.21	0.90	0.03	*Ruminococcaceae* (f)
OTU49	0.25	0.06	0.22	0.30	0.29	0.19	0.12	0.29	0.09	0.16	0.80	0.59	0.30	0.66	0.03	*Ruminococcaceae* (f)
OTU50	0.12	0.17	0.16	0.34	0.09	0.15	0.13	0.20	0.07	0.09	0.26	0.74	0.10	0.02	0.49	*Clostridiales* (o)
OTU51	0.37	0.10	0.18	0.14	0.28	0.08	0.18	0.20	0.06	<0.01	0.75	0.69	<0.01	0.06	0.01	*Oscillospira* (g)
OTU56	0.18	0.15	0.13	0.18	0.27	0.10	0.09	0.22	0.05	0.07	0.71	0.47	0.06	0.61	0.01	*Clostridiales* (o)
OTU57	0.09	0.14	0.20	0.16	0.18	0.10	0.23	0.08	0.04	0.09	0.98	0.20	0.68	0.76	0.20	*Lachnospiraceae* (f)
OTU65	0.18	0.07	0.11	0.10	0.16	0.10	0.11	0.12	0.04	0.18	0.69	0.92	0.04	0.21	0.13	*Ruminococcaceae* (f)

OTU67	0.14	0.19	0.09	0.15	0.13	0.08	0.05	0.11	0.03	0.16	0.03	0.45	0.43	0.49	0.21	*Ruminococcaceae* (f)
OTU69	0.04	0.12	0.09	0.16	0.05	0.08	0.11	0.12	0.04	0.18	0.75	0.85	0.06	0.04	0.84	*Clostridiales* (o)
OTU70	0.21	0.06	0.13	0.07	0.16	0.07	0.12	0.08	0.05	0.07	0.72	0.95	0.02	0.08	0.32	*Enterobacteriaceae* (f)
OTU71	0.17	0.07	0.07	0.13	0.17	0.08	0.08	0.13	0.04	0.02	0.93	1.00	0.01	0.24	<0.01	*Lachnospiraceae* (f)
OTU73	0.20	0.13	0.08	0.09	0.10	0.06	0.08	0.08	0.04	0.18	0.09	0.48	0.03	0.06	0.26	*Enterobacteriaceae* (f)
OTU75	0.06	0.12	0.14	0.08	0.07	0.10	0.13	0.07	0.03	0.14	0.79	0.97	0.15	0.74	0.02	*Lachnospiraceae* (f)
OTU76	0.06	0.10	0.13	0.10	0.10	0.11	0.11	0.08	0.02	0.19	0.94	0.33	0.10	0.46	0.04	*Coprococcus* (g)
OTU77	0.16	0.07	0.09	0.05	0.18	0.06	0.05	0.06	0.03	<0.01	0.73	0.61	<0.01	<0.01	0.03	*Clostridium* (g)
OTU79	0.13	0.09	0.07	0.15	0.09	0.07	0.07	0.12	0.03	0.19	0.24	0.95	0.67	0.44	0.05	*Clostridiales* (o)
OTU87	0.13	0.05	0.07	0.05	0.11	0.06	0.07	0.08	0.02	0.08	0.80	0.85	0.01	0.09	0.11	*Ruminococcaceae* (f)
OTU91	0.06	0.06	0.19	0.07	0.08	0.05	0.07	0.05	0.03	0.06	0.20	0.13	0.54	0.56	0.18	*Lachnospiraceae* (f)
OTU93	0.10	0.15	0.05	0.07	0.09	0.04	0.05	0.05	0.03	0.20	0.04	0.19	0.21	0.07	0.85	*Ruminococcaceae* (f)
OTU94	0.04	0.08	0.11	0.05	0.06	0.08	0.11	0.05	0.03	0.11	0.82	0.98	0.15	0.58	0.03	*Lachnospiraceae* (f)
OTU105	0.04	0.06	0.05	0.10	0.04	0.04	0.03	0.08	0.02	0.08	0.31	0.99	0.23	0.04	0.23	*Ruminococcaceae* (f)
OTU107	0.03	0.02	0.14	0.04	0.08	0.06	0.14	0.06	0.05	0.13	0.43	0.96	0.51	0.51	0.25	[*Ruminococcus*] (g)
OTU117	0.01	0.08	0.10	0.03	0.02	0.07	0.10	0.04	0.03	0.03	0.93	0.97	0.02	0.27	<0.01	*Blautia producta* (s)
OTU119	0.07	0.04	0.06	0.03	0.07	0.04	0.06	0.05	0.01	0.01	0.68	0.85	0.01	0.05	0.40	*Oscillospira* (g)
Firm./Proteo.	9.08	17.74	14.93	13.31	10.28	9.63	8.70	24.09	4.45	0.22	0.85	0.14	0.17	0.08	0.65	

[1]Data are presented as least square means ± standard error of the mean (SEM); n=10 per treatment group. Only values for OTUs with a relative abundance > 0.05% which were differently affected ($p < 0.05$) by treatments are presented. Firm./Proteo., *Firmicutes*-to-*Proteobacteria* ratio.

[2]*P*-values for orthogonal contrasts to test linear (lin.) and quadratic (quad.) relationships between control feeding and the three increasing levels of DON as well as the overall difference of 0 DON versus all DON groups (0 vs. DON).

[a,b] DON × LPS interaction: Least square means of OTUs with no common superscripts differ significantly between groups; $p < 0.05$.

Supplementary Table S3. List of the most abundant operational taxonomic units (OTUs).[1]

OTU	Accession number	Result BLAST Greengenes	Similarity, %
OTU1	ABDU01000080.2	*Clostridium perfringens C str. JGS1495*	83.4
	NZ_ABDU01000080.2		
OTU2	GQ222401.1	*Escherichia coli str. FUA 1062*	96.0
	HM486679.1	*E.coli str. PGB 01*	
	GQ222391.1	*E. coli str. FUA 1070*	
	HM146924.1	*Shigella sp. str. RSI091219*	
	GU968183.1	*E. coli str. 46*	
	HQ169122.1	*E. coli str. FUA 1241*	
	ADTR01000016.1	*E. coli str. MS 21-1*	
	ADWV01000001.1	*E. coli str. MS 107-1*	
	GQ222389.1	*E. coli str. FUA 1036*	
	FJ839361.1	*Shigella flexneri str. G3*	
OTU3	ABDU01000080.2	*Clostridium perfringens C str. JGS1495*	83.4
	NZ_ABDU01000080.2		
OTU11	ABDU01000080.2	*Clostridium perfringens C str. JGS1495*	83.6
	NZ_ABDU01000080.2		
OTU12	NC_011898.1	*Clostridium cellulolyticum H10 str. H10; ATCC 35319*	83.2
OTU13	DQ279736.1	*Clostridium aldenense str. RMA 9741*	91.8
OTU15	ACFX02000046.1	*Clostridium sp. str. M62/1;*	93.2
	NZ_ACFX01000080.1		
	NZ_ACFX02000046.1		
	FP929060.1	*Clostridiales sp. SM4/1*	
OTU16	FJ805840.2	*Clostridium sp. str. BS-1*	90.6
OTU18	NR_027558.1	*Anaerotruncus colihominis str. WAL 14565; DSM 17241*	95.6
	AJ315980.1	*Anaerotruncus colihominis str. 14565*	
	DQ002932.1	*Anaerotruncus colihominis str. HKU19*	
	ABGD02000031.1	*Anaerotruncus colihominis str. DSM 17241*	
	NZ_ABGD02000021.1		
	NZ_ABGD02000031.1		
	NZ_ABGD02000032.1		
OTU22	NR_027558.1	*Anaerotruncus colihominis str. WAL 14565; DSM 17241; HKU19*	95.4
	AJ315980.1	*Anaerotruncus colihominis str. 14565*	

	DQ002932.1	*Anaerotruncus colihominis str. HKU19*	
	ABGD02000031.1	*Anaerotruncus colihominis str. DSM 17241*	
	NZ_ABGD02000021.1		
	NZ_ABGD02000031.1		
	NZ_ABGD02000032.1		
OTU24	EF031543.1	*Coprococcus eutactus str. ATCC 27759*	80.5
	NZ_ABEY02000009.1		
	NZ_ABEY02000015.1		
	NZ_ABEY02000028.1		
	NZ_ABEY02000025.1		
	EU728700.1	*Coprococcus sp. str. DJF_B005*	
OTU28	AB491208.1	*Clostridium sp. str. YIT 12070*	90.3
OTU29	AY487928.1	*Acetanaerobacterium elongatum str. Z7*	89.4
OTU41	AB571656.1	*Blautia coccoides str. JCM 1395*	93.0
	AB196512.1	*Ruminococcus productus str. M-2*	
	AY937379.1	*Ruminococcus productus str. SECO-Mt75m3*	
	EF025906.1	*Clostridium coccoides str. 8F*	
	GU124472.1	*Blautia sp. Str. Ser8*	
OTU42	EU815224.1	*Clostridium sp. str. NML 04A032*	91.5
	NZ_AAXG02000037.1	*Bacteroides capillosus str. ATCC 29799*	
	NZ_AAXG02000048.1		
OTU43	ACFX02000046.1	*Clostridium sp. str. M62/1*	94.1
	FP929060.1	*Clostridiales sp. SM4/1*	
OTU47	NZ_AAXG02000037.1	*Bacteroides capillosus str. ATCC 29799*	94.7
	NZ_AAXG02000048.1		
OTU48	Z49863.1	*Sporobacter termitidis str. SYR*	89.8
OTU49	Z49863.1	*Sporobacter termitidis str. SYR*	90.2
OTU50	NR_024919.1	*Clostridium frigidicarnis str. SPL77A; DSM 12271*	85.0
	AF069742.1	*Clostridium frigidicarnis str. SPL77A*	
OTU51	NZ_AAXG02000037.1	*Bacteroides capillosus str. ATCC 29799*	93.6
	NZ_AAXG02000048.1		
OTU56	DQ279736.1	*Clostridium aldenense str. RMA 9741*	91.8
OTU57	NR_026100.1	*Clostridium celerecrescens str. DSM 5628*	92.2
	DQ677005.1	*Clostridium sp. Iso-A1*	

	DQ677019.1	*Desulfotomaculum sp. Iso-W2*	
	AB277863.1	*Clostridium sp. Str. T1'2*	
	AB277866.1	*Clostridium sp. Str. U42*	
	AM884908.1	*Clostridium sp. Str. ADS23*	
	EU869245.1	*Clostridium sp. Str. CM-C99*	
	GU195653.1	*Clostridium sp. Str. ZP3*	
	NR_026409.1	*Desulfomaculum guttoideum str. DSM 4024*	
	FM994938.1	*Clostridium celerecrescens str. HP2*	
OTU65	AB491208.1	*Clostridium sp. str. YIT 12070*	91.1
OTU67	L34618.1	*Eubacterium desmolans*	92.4
OTU69	AY312403.2	*Alkalibacter saccharofermentans str. Z-79820*	83.5
OTU70	HM007577.1	*Salmonela serovar Enteritidis subsp. Enterica str. 13*	96.0
	GU370901.1	*Salmonella enterica str. AB7*	
	HM007581.1	*Salmonella serovar Typhimurium subsp. Enterica str. 5275*	
	FQ312003.1	*Salmonella subsp. enterica serovar Typhimurium str. SL1344*	
OTU71	FP929060.1	*Clostridiales sp. SM4/1*	92.8
	ACFX02000046.1	*Clostridium sp. str. M62/1*	
	NZ_ACFX01000080.1		
	NZ_ACFX02000046.1		
OTU73	U92193.1	*Salmonella enterica serovar Bovis-morbificans subsp. morbificans str. Sbm1 subsp*	98.9
	U88546.1	*Salmonella paratyphi*	
	U92196.1	*Salmonella subsp. enterica serovar Bareilly str. Sb1 subsp*	
	U92195.1	*Salmonella enterica subsp. houtenae serovar Houten str. Sh1 subsp.*	
OTU75	CP002109.1	*Clostridium saccharolyticum str. WM1*	92.5
	NR_026494.1	*Clostridium saccharolyticum WM1 str. WM1, DSM 2544*	
	AM884908.1	*Clostridium sp. str. ADS23*	
	EU869245.1	*Clostridium sp. Str. CM-C99*	
	AF520990.1	*Clostridium sp. Str. TDO*	
OTU76	AY960565.1	*Clostridium sp. str. ID11*	93.4
OTU77	FJ808599.1	*Clostridium sp. str. 4-1*	86.6
	FJ808600.1	*Clostridium sp. str. 4-2a*	
OTU79	ACFX02000046.1	*Clostridium sp. str. M62/1*	90.5
	NZ_ACEFX01000080.1		

	NZ_ACEFX020000046.1		
	FP929060.1	*Clostridiales sp. SM4/1*	
OTU87	AB491208.1	*Clostridium sp. str. YIT 12070*	91.3
OTU91	ACFX02000046.1	*Clostridium sp. str. M62/1*	93.6
	NZ_ACFX01000080.1		
	NZ_ACFX02000046.1		
	FP929060.1	*Clostridiales sp. SM4/1*	
OTU93	L34618.1	*Eubacterium desmolans*	96.2
OTU94	ACFX02000046.1	*Clostridium sp. str. M62/1*	91.9
	NZ_ACFX01000080.1		
	NZ_ACFX02000046.1		
	FP929060.1	*Clostridiales sp. SM4/1*	
OTU105	AY487923.1	*Acetanaerobacterium elongatum str. Z7*	90.1
	AY518589.1	*Acetanaerobacterium elongatum str. Z1*	
OTU107	DQ057463.1	*str. ic1311*	94.7
OTU117	AB571656.1	*Blautia coccoides str. JCM 1395*	92.6
	AB196512.1	*Ruminococcus productus str. M-2*	
	AY937379.1	*Ruminococcus productus str. SECO-Mt75m3*	
	EF451052.1	*Ruminococcus sp. str. END-1*	
	EF025906.1	*Clostridium coccoides str. 8F*	
	GU124472.1	*Blautia sp. Str. Ser8*	
OTU119	EU815224.1	*Clostridium sp. str. NML 04A032*	91.2
	NZ_AAXG02000037.1	*Bacteroides capillosus str. ATCC 29799*	
	NZ_AAXG02000048.1		

[1]The best type strain hits (Greengenes database), its accession numbers and the similarity percentage are listed. Only OTUs with a relative abundance > 0.05% that were differently affected ($p < 0.05$) by the treatments are presented.

Supplementary Table S4. Relative abundance (%) of Kyoto Encyclopedia of Genes and Genomes (KEGG) pathways in cecal digesta of broiler chickens fed diets with increasing levels of deoxynivalenol (DON) and with or without oral lipopolysaccharide challenge (LPS) 1 day prior to slaughter.[1]

	no LPS				LPS					Fixed effect, P-values			Contrasts, P-values[1]		
DON (mg/kg feed)	0	2.5	5	10	0	2.5	5	10	SEM	DON	LPS	DON × LPS	0 vs. DON	lin.	quad.
**<u>Cellular Processes</u>**															
Cell Motility															
Bacterial motility proteins	1.35	1.64	1.49	1.74	1.38	1.63	1.63	1.73	0.14	0.08	0.71	0.95	0.02	0.03	0.66
Flagellar assembly	0.59	0.73	0.67	0.78	0.61	0.74	0.74	0.79	0.07	0.09	0.62	0.96	0.02	0.03	0.62
Bacterial chemotaxis	0.57	0.70	0.64	0.75	0.59	0.67	0.67	0.74	0.06	0.05	0.95	0.95	0.02	0.01	0.89
**<u>Environmental Information Processing</u>**															
Membrane Transport															
Bacterial secretion system	0.64	0.60	0.60	0.58	0.62	0.62	0.64	0.58	0.02	0.07	0.58	0.39	0.09	0.02	0.48
Signal Transduction															
Phosphatidylinositol signaling system	0.08	0.07	0.08	0.07	0.08	0.08	0.08	0.07	<0.01	0.17	0.35	0.91	0.09	0.048	0.86
**<u>Genetic Information Processing</u>**															
Folding, Sorting and Degradation															
Protein processing in endoplasmic reticulum	0.06	0.06	0.06	0.07	0.06	0.07	0.06	0.07	<0.01	0.17	0.93	1.00	0.04	0.06	0.53

Replication and Repair															
Base excision repair	0.44	0.40	0.42	0.40	0.43	0.42	0.42	0.41	0.01	0.09	0.79	0.89	0.02	0.03	0.59
Transcription															
Transcription machinery	0.96	1.03	1.00	1.06	0.96	0.99	1.00	1.05	0.04	0.15	0.56	0.96	0.07	0.04	0.93
**<u>Metabolism</u>**															
Amino Acid Metabolism															
Glycine, serine and threonine metabolism	0.77	0.73	0.75	0.72	0.77	0.74	0.73	0.72	0.02	0.04	0.65	0.89	0.01	0.01	0.60
Histidine metabolism	0.63	0.60	0.61	0.60	0.62	0.60	0.61	0.60	0.01	0.16	0.86	0.87	0.03	0.12	0.29
Phenylalanine metabolism	0.18	0.18	0.19	0.18	0.18	0.18	0.19	0.18	<0.01	0.10	0.39	0.92	0.05	0.22	0.04
Tryptophan metabolism	0.16	0.13	0.14	0.12	0.16	0.15	0.14	0.12	0.02	0.16	0.58	0.94	0.06	0.04	0.99
Lysine degradation	0.15	0.13	0.13	0.11	0.15	0.14	0.13	0.12	0.01	0.14	0.81	0.96	0.06	0.03	0.99
Biosynthesis of Other Secondary Metabolites															
Streptomycin biosynthesis	0.26	0.28	0.28	0.29	0.28	0.28	0.28	0.28	0.01	0.02	0.67	0.17	<0.01	<0.01	0.26
Novobiocin biosynthesis	0.13	0.13	0.13	0.13	0.13	0.13	0.13	0.13	0.002	0.07	0.91	0.74	0.01	0.05	0.31
Tropane, piperidine and pyridine alkaloid biosynthesis	0.11	0.11	0.11	0.11	0.11	0.11	0.11	0.10	0.002	0.09	0.37	0.71	0.04	0.02	0.96
Isoquinoline alkaloid biosynthesis	0.03	0.04	0.04	0.04	0.04	0.04	0.04	0.04	0.002	0.02	0.59	0.41	<0.01	0.02	0.16
Flavone and flavonol	0.003	0.002	0.002	0.003	0.004	0.002	0.002	0.003	0.001	0.12	0.70	0.63	0.11	0.72	0.02

biosynthesis															
Carbohydrate Metabolism															
Citrate cycle (TCA cycle)	0.55	0.56	0.58	0.56	0.55	0.59	0.59	0.57	0.01	0.03	0.28	0.81	0.02	0.17	0.01
Propanoate metabolism	0.57	0.56	0.57	0.55	0.57	0.57	0.56	0.55	0.01	0.10	0.93	0.95	0.09	0.02	0.57
Glyoxylate and dicarboxylate metabolism	0.53	0.48	0.50	0.46	0.53	0.49	0.50	0.46	0.03	0.06	0.98	0.99	0.02	0.02	0.79
Energy Metabolism															
Nitrogen metabolism	0.69	0.73	0.71	0.74	0.70	0.75	0.72	0.74	0.02	0.06	0.63	0.97	0.02	0.08	0.40
Enzyme Families															
Peptidases	1.81	1.79	1.80	1.79	1.82	1.78	1.77	1.79	0.02	0.23	0.52	0.75	0.04	0.19	0.15
Glycan Biosynthesis and Metabolism															
Glycosyltransferases	0.28	0.26	0.26	0.26	0.27	0.28	0.27	0.26	0.01	0.18	0.34	0.35	0.15	0.04	0.61
Lipopolysaccharide biosynthesis proteins	0.22	0.18	0.19	0.16	0.22	0.22	0.21	0.16	0.03	0.20	0.42	0.88	0.15	0.047	0.62
N-Glycan biosynthesis	0.01	0.02	0.01	0.02	0.01	0.02	0.02	0.02	0.004	0.07	0.98	0.99	0.01	0.02	0.56
Glycosaminoglycan degradation	0.01	0.01	0.01	0.01	0.02	0.01	0.01	0.01	0.002	0.22	0.51	0.70	0.04	0.07	0.31
Lipid Metabolism															
Fatty acid biosynthesis	0.53	0.51	0.50	0.50	0.52	0.50	0.50	0.49	0.01	0.08	0.37	0.88	0.01	0.02	0.41
Fatty acid metabolism	0.27	0.25	0.26	0.24	0.27	0.26	0.26	0.24	0.01	0.08	0.52	0.87	0.06	0.02	0.70
Biosynthesis of	0.15	0.14	0.14	0.13	0.15	0.15	0.15	0.13	0.01	0.15	0.79	0.96	0.13	0.04	0.59

unsaturated fatty acids															
Sphingolipid metabolism	0.13	0.13	0.12	0.13	0.14	0.12	0.11	0.13	0.01	0.18	0.48	0.32	0.18	0.67	0.04
Synthesis and degradation of ketone bodies	0.04	0.03	0.03	0.03	0.04	0.03	0.03	0.03	0.01	0.09	0.99	0.91	0.01	0.03	0.28
Metabolism of Cofactors and Vitamins															
Porphyrin and chlorophyll metabolism	0.79	0.90	0.88	0.94	0.85	0.86	0.86	0.93	0.04	0.06	0.89	0.68	0.02	0.01	0.95
Thiamine metabolism	0.50	0.54	0.52	0.55	0.51	0.53	0.52	0.55	0.02	0.12	0.87	0.99	0.04	0.04	0.83
Nicotinate and nicotinamide metabolism	0.44	0.44	0.44	0.43	0.44	0.44	0.45	0.43	0.01	0.18	0.94	0.98	0.46	0.70	0.04
Metabolism of Other Amino Acids															
Glutathione metabolism	0.20	0.18	0.18	0.17	0.20	0.20	0.19	0.17	0.01	0.22	0.43	0.83	0.10	0.04	0.90
D-Alanine metabolism	0.11	0.10	0.10	0.10	0.11	0.11	0.10	0.10	0.001	0.01	0.66	0.28	<0.01	<0.01	0.10
Metabolism of Terpenoids and Polyketides															
Polyketide sugar unit biosynthesis	0.16	0.17	0.17	0.17	0.17	0.17	0.17	0.17	0.004	0.02	0.74	0.32	<0.01	0.01	0.46
Tetracycline biosynthesis	0.18	0.16	0.16	0.15	0.17	0.16	0.16	0.15	0.01	0.05	0.56	0.94	0.01	0.02	0.53
Biosynthesis of ansamycins	0.13	0.14	0.14	0.14	0.13	0.13	0.14	0.14	0.01	0.07	0.28	0.58	0.02	0.01	0.48
Biosynthesis of	0.04	0.03	0.03	0.02	0.04	0.04	0.03	0.03	0.01	0.22	0.51	0.87	0.11	0.046	0.83

siderophore group nonribosomal peptides															
Xenobiotics Biodegradation and Metabolism															
Drug metabolism - other enzymes	0.33	0.32	0.32	0.32	0.33	0.32	0.31	0.32	0.01	0.24	0.44	0.61	<0.05	0.15	0.15
Chloroalkane and chloroalkene degradation	0.23	0.25	0.26	0.26	0.24	0.26	0.25	0.26	0.01	<0.01	0.57	0.36	<0.01	<0.01	0.04
Benzoate degradation	0.27	0.24	0.25	0.24	0.26	0.24	0.25	0.24	0.01	0.01	0.84	0.72	<0.01	0.02	0.25
Nitrotoluene degradation	0.09	0.12	0.12	0.13	0.10	0.12	0.12	0.12	0.01	0.02	0.78	0.97	<0.01	0.01	0.21
Caprolactam degradation	0.04	0.03	0.03	0.03	0.04	0.04	0.04	0.03	0.01	0.16	0.48	0.94	0.12	0.04	0.64
Fluorobenzoate degradation	0.004	0.003	0.003	0.002	0.004	0.004	0.004	0.002	0.001	0.17	0.45	0.81	0.15	0.04	0.55
**Unclassified**															
Cellular Processes and Signaling															
Sporulation	1.08	1.22	1.21	1.26	1.13	1.19	1.16	1.26	0.05	0.03	0.81	0.79	0.01	0.01	0.76
Other ion-coupled transporters	1.22	1.13	1.19	1.11	1.23	1.17	1.16	1.10	0.05	0.07	0.96	0.93	0.03	0.02	0.89
Other transporters	0.31	0.28	0.29	0.28	0.30	0.29	0.29	0.28	0.01	0.01	0.88	0.67	<0.01	<0.01	0.76
Pores ion channels	0.27	0.24	0.24	0.23	0.25	0.26	0.26	0.23	0.02	0.15	0.51	0.50	0.11	0.04	0.67
Genetic Information Processing															
Translation proteins	0.99	0.95	0.96	0.94	0.97	0.95	0.95	0.94	0.01	<0.01	0.54	0.72	<0.01	<0.01	0.14
Protein folding and	0.58	0.56	0.56	0.55	0.59	0.58	0.56	0.55	0.01	0.14	0.53	0.91	0.05	0.02	0.86

associated processing															
Restriction enzyme	0.18	0.20	0.19	0.21	0.17	0.20	0.19	0.20	0.01	0.12	0.87	0.99	0.03	0.05	0.66
Metabolism															
Others	0.96	0.92	0.94	0.91	0.96	0.94	0.93	0.93	0.02	0.17	0.46	0.83	0.03	0.07	0.33
Poorly Characterized															
General function prediction only	3.65	3.57	3.58	3.56	3.61	3.58	3.60	3.56	0.02	0.01	0.97	0.57	<0.01	<0.01	0.25

[1]Data are presented as least square means ± standard error of the mean (SEM); n=10 per treatment group. Only values for KEGG pathways that were differently affected ($p < 0.05$) by treatments are presented.

[2]*P*-values for orthogonal contrasts to test linear (lin.) and quadratic (quad.) relationships between control feeding and the three increasing levels of DON as well as the overall difference of 0 DON versus all DON groups (0 vs. DON).
